# Transgenic Overexpression of Tcfap2c/AP-2gamma Results in Liver Failure and Intestinal Dysplasia

**DOI:** 10.1371/journal.pone.0022034

**Published:** 2011-07-13

**Authors:** Daniel Holl, Peter Kuckenberg, Tatiana Woynecki, Angela Egert, Astrid Becker, Sebastian Huss, Dirk Stabenow, Andreas Zimmer, Percy Knolle, René Tolba, Hans-Peter Fischer, Hubert Schorle

**Affiliations:** 1 Department of Developmental Pathology, Institute of Pathology, University of Bonn Medical School, Bonn, Germany; 2 Institute of Molecular Psychiatry, University of Bonn, Bonn, Germany; 3 Institute of Pathology, University of Bonn Medical School, Bonn, Germany; 4 Institute of Experimental Immunology, University of Bonn, Bonn, Germany; 5 Institute for Laboratory Animal Science and Experimental Surgery, Aachen University, Aachen, Germany; Baylor College of Medicine, United States of America

## Abstract

**Background:**

The transcription factor Tcfap2c has been demonstrated to be essential for various processes during mammalian development. It has been found to be upregulated in various undifferentiated tumors and is implicated with poor prognosis. Tcfap2c is reported to impinge on cellular proliferation, differentiation and apoptosis. However, the physiological consequences of Tcfap2c-expression remain largely unknown.

**Methodology/Principal Findings:**

Therefore we established a gain of function model to analyze the role of Tcfap2c in development and disease. Induction of the transgene led to robust expression in all tissues (except brain and testis) and lead to rapid mortality within 3–7 days. In the liver cellular proliferation and apoptosis was detected. Accumulation of microvesicular lipid droplets and breakdown of major hepatic metabolism pathways resulted in steatosis. Serum analysis showed a dramatic increase of enzymes indicative for hepatic failure. After induction of Tcfap2c we identified a set of 447 common genes, which are deregulated in both liver and primary hepatocyte culture. Further analysis showed a prominent repression of the cytochrome p450 system, PPARA, Lipin1 and Lipin2. These data indicate that in the liver Tcfap2c represses pathways, which are responsible for fatty acid metabolism. In the intestine, Tcfap2c expression resulted in expansion of Sox9 positive and proliferative active epithelial progenitor cells resulting in dysplastic growth of mucosal crypt cells and loss of differentiated mucosa.

**Conclusions:**

The transgenic mice show that ectopic expression of Tcfap2c is not tolerated. Due to the phenotype observed, iTcfap2c-mice represent a model system to study liver failure. In intestine, Tcfap2c induced cellular hyperplasia and suppressed terminal differentiation indicating that Tcfap2c serves as a repressor of differentiation and inducer of proliferation. This might be achieved by the Tcfap2c mediated activation of Sox9 known to be expressed in intestinal and hepatic stem/progenitor cell populations.

## Introduction

The family of Activator Protein-2 (AP-2) transcription factors is highly conserved in mice (Tcfap2a–e) and humans (TFAP2A–E). Tcfap2a, as first protein of the transcription factor family was isolated from HeLa cells in 1988 [1], followed by the isoforms Tcfap2b [2], Tcfap2c [3], Tcfap2d [4] and Tcfap2e [5]. AP-2 proteins have been demonstrated to modulate various signaling pathways during development, cell growth, differentiation and apoptosis [6], [7], [8], [9], [10]. AP-2 proteins are known to orchestrate the balance between cellular growth and differentiation and are essential for maintaining cellular homeostasis [11].

During murine development Tcfap2c expression is restricted spatiotemporal to facial and limb mesenchyme, extraembryonic tissue, primordial germ cells, peripheral nervous system, neural-crest cells, dorsal telencephalon and various epithelia of the developing embryo [12]. In adults, its expression is limited to the developing breast, where loss of Tcfap2c blocks branching morphogenesis of the mammary gland before puberty [13].

Deficiency of Tcfap2c causes early embryonic lethality on day 7.5 dpc due to a defect in the extraembryonic compartment. Tcfap2c is required to maintain the extraembryonic lineages and the undifferentiated state of trophoblast stem cells [9], [14]. Further, conditional deletion of Tcfap2c leads to loss of primordial germ cells short after specification on day 8.0 dpc caused by de-repression of somatic differentiation of PGC [15]. Additionally, conditional deletion of Tcfap2c affects neurogenic basal progenitor fate at mid-neurogenesis in the developing cortex [16].

Many studies have demonstrated TFAP2C expression in a variety of solid tumors in humans. TFAP2C is reported to be upregulated in breast cancer [17], [18], [19], [20], in squamous cell carcinoma (SCC) [21], in germ cell tumors and ovarian cancer [22], [23]. In mammary cancer, TFAP2C expression results in increased progression of the tumors [24]. In general, Tcfap2c is often found in undifferentiated cells or progenitor cell populations and represses differentiation during development as well as in cancer. Several studies demonstrated a variety of target genes for TFAP2C in breast cancer tissue and cell lines [25], [26], [27], [28], [29]. In breast cancer TFAP2C transactivates the expression of HER-2/neu (ErbB2) [30] and estrogen receptor genes [29]. TFAP2C has been shown to regulate the cyclin-dependent kinase inhibitor (p21CIP1/CDKN1A) in breast cancer cell lines, but data are controversial. While in MDA MB-231 cells TFAP2C activates p21 and represses proliferation, in MCF7 cells TFAP2C suppresses p21 [28], [31]. Hence ectopic transgene induction is likely to have major effects and also mimics the reactivation of expression observed in some solid tumor types. Better knowledge on the set of Tcfap2c target genes and affected pathways in different tissues would be beneficial for the interpretation of the results. Therefore we established a mouse model, in which Tcfap2c can be activated in an inducible and reversible manner in somatic tissues, taking advantage of the tetracycline-dependent regulatory system.

## Results

### Generation of dox-inducible Tcfap2c transgenic mice

**Figure 1:**
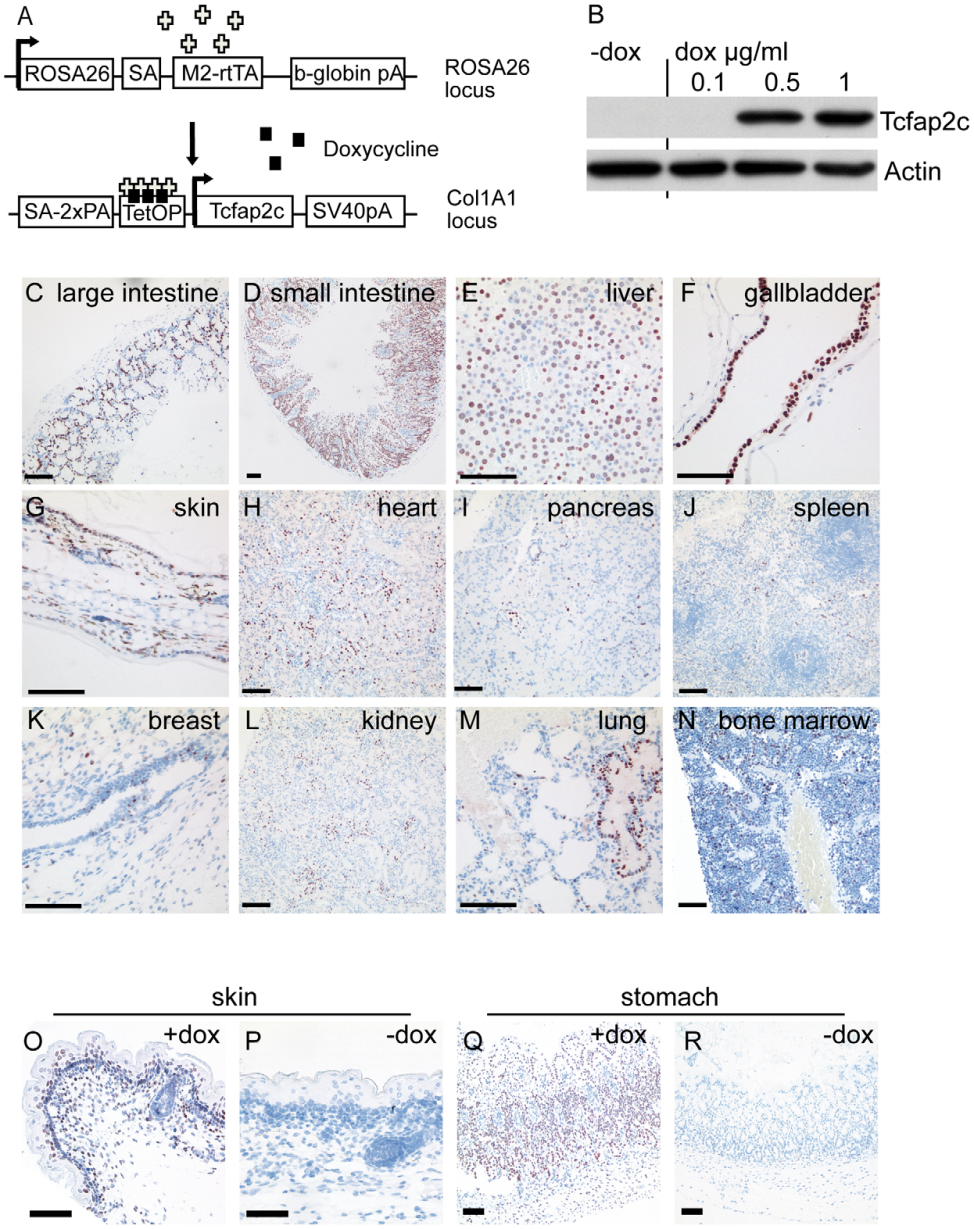
Generation of iTcfap2c-mice. Schematic representation of transgenes used to produce Tcfap2c-inducible ES cells and mice. ES cells containing M2-rtTA expressed under control of the Rosa26 promoter were retargeted at the 3′ UTR of the Col1a1 locus by insertion of the murine Tcfap2c-cDNA under control of the tetracycline responsive element (TetOP) (pA, polyadenylation signal). (B) Western blot analysis of doxycyline (dox) dependent Tcfap2c induction in transgenic ES cells. (C–N) Immunohistochemical detection of Tcfap2c protein on sections from iTcfap2c-mice after dox-induction. Lack of Tcfap2c expression in untreated iTcfap2c mice in skin (P) and stomach (R) compared to dox induced iTcfap2c tissue (O,Q). Scale bars = 100 µm.

We had previously established mouse embryonic stem cell (ESC) lines harboring the Tcfap2c-cDNA 3′ of the Col1a1 locus under control of a tetracycline responsive element (Col1a1::TetO-Tcfap2c). There, the reverse tetracycline transactivator (M2-rtTA) is integrated in the ROSA26 locus (ROSA26::rtTA) and expressed by its endogenous promotor (Fig.1 A) [14]. The ESC-lines displayed doxycycline-dependent induction of transgenic Tcfap2c (Fig.1 B) after 48 h and were used to generate transgenic mice. Mice harboring both, the Col1a1::TetO-Tcfap2c and ROSA26::rtTA transgenes named inducible Tcfap2c (iTcfap2c) animals were selected for experiments and littermates were used as controls.

### Doxycycline dependent expression of Tcfap2c in adult mice

To determine the levels of Tcfap2c-induction in various tissues of adult mice, we analyzed animals that were given doxycycline (dox) by drinking water for 5 days. Tissues were collected and analyzed by immunohistochemistry. Highest levels of Tcfap2c were detected in gastrointestinal tract, liver, gallbladder, skin and uterus (Fig.1 C–G). Lower levels were found in heart, pancreas, spleen, breast, kidney, lung, and bone marrow (Fig.1 H–N).

Evaluation of the distribution of transgene expression revealed that the expression varied within the tissues. Strong and uniform expression was detected in hepatocytes of the liver, cells of the extrahepatic bile ducts, intestinal crypt- and villus-cells, stomach, epithelial cells of the epidermis and the endometrial cells of the uterus (Table S1). In brain and testis tissue, no induction was detected. This might be the result of poor dox-penetration due to the blood–brain and blood-testis barrier. Using RT-PCR, transgene expression in liver, kidney and intestine was verified (not shown). iTcfap2c-mice that were not exposed to dox did not show any detectable expression of the Tcfap2c transgene (Fig.1 P, R, compared to O, Q). Hence, these data show that we had established a dox-dependent conditional transgenic system for the induction of Tcfap2c.

### Induction of Tcfap2c causes morbidity

**Figure 2:**
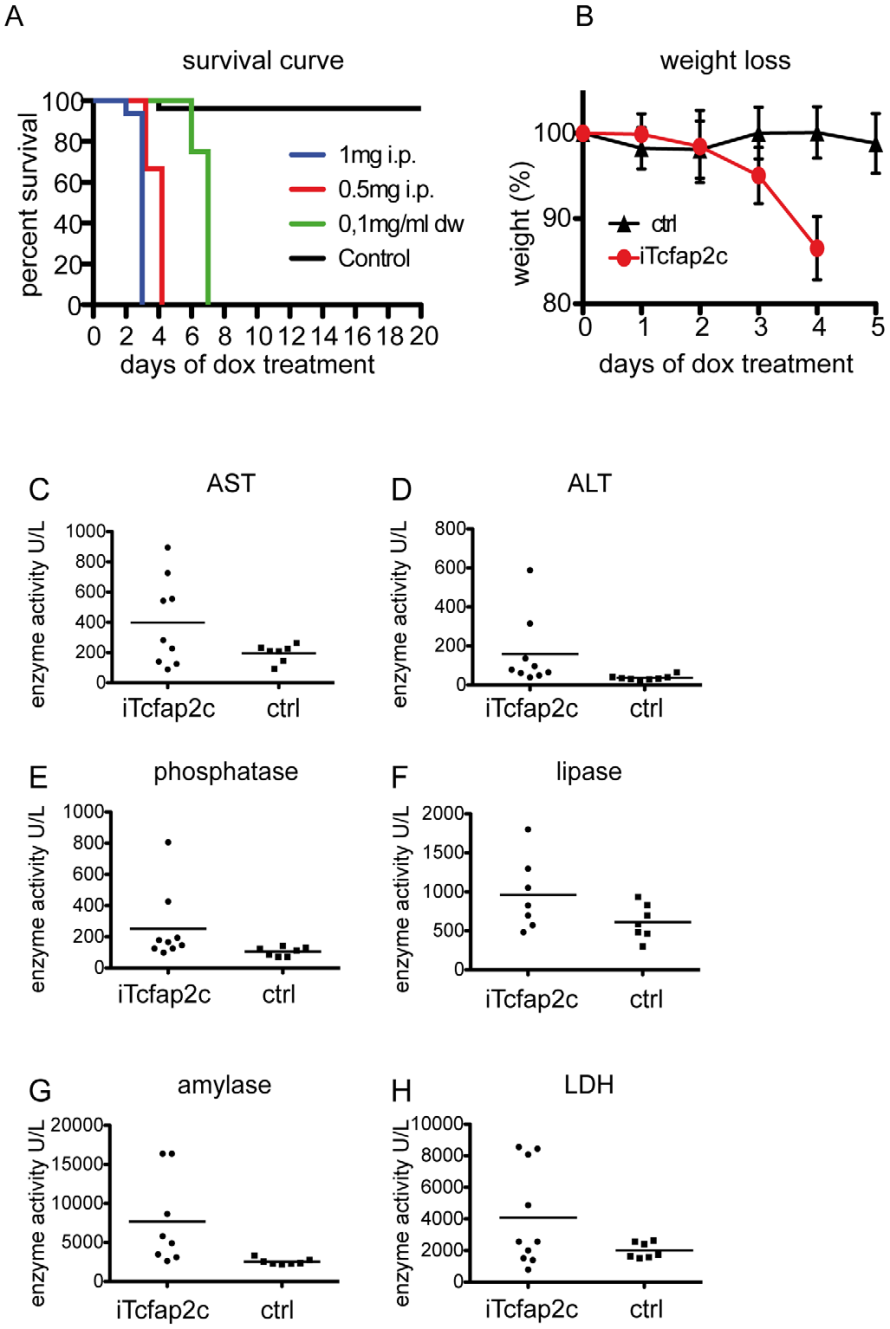
iTcfap2c-mice show strong morbidity in a dox-dependent manner. (A) Kaplan-Meier plot showing survival after dox treatment. Green line: iTcfap2c-mice receiving dox via drinking water (dw) (0.1 mg/ml, n = 6). Blue and red line: iTcfap2c-mice receiving dox via intra peritoneal injection (i.p.) (blue: 1 mg/day, n = 33), (red: 0.5 mg/day n = 12). Black line: control mice (0.1 mg/ml dox by in dw for 20 days). (B) Weight of iTcfap2c (red) and ctrl animals (black), which received 0.5 mg dox i.p. per day (n = 12). (C–H) Biochemical blood serum analysis. Enzyme activity was measured as the concentration of substrate converted per minute (1 enzyme unit = 1 µmol substrate/min) in units/ liter. iTcfap2c (n = 9) and ctrl animals (n = 7) were induced with 1 mg/ml dox per day. Blood samples were collected the third day.

Next, we assessed the physiological consequences of transgene induction. After administration of dox (0.1 mg/ml) in the drinking water, iTcfap2c-animals became lethargic lost up to 15% of their weight (Fig.2 B) showed signs of dehydration and died within 6-7 days (Fig.2 A). Administration of dox via intraperintoneal (i.p.) injection lead to death within 4 (0.5 mg/d, n = 6) or 3 (1 mg/d, n = 17) days. Control animals treated 20 days with dox in the drinking water or by i.p.-injection, showed no signs of morbidity (Fig.2 A).

To identify the cause of lethality, we next analyzed metabolic parameters in the blood/serum. Therefore, iTcfap2c (n = 9) and control animals (n = 7) were treated with dox (0.5 mg/d) three times (0, 24 and 48 h) and blood was collected after 52 h. The iTcfap2c-mice showed elevated levels of aspartate transaminase, alanine transaminase and alkaline phosphatase (Fig. 2 C-E), enzymes indicative for acute liver failure. Furthermore serum levels of lipase and amylase were increased. These are markers for pancreatic injury (Fig.2 F–G). Also, five mice showed increased lactate dehydrogenase levels, a marker of general tissue breakdown (Fig.2 H). The fact, that we did not detect an increase of CD45 common leukocyte antigen staining in liver and intestine, indicated that transgenic expression of Tcfap2c did not result in the induction inflammatory processes (not shown). These data suggest that induction of Tcfap2c leads to liver failure. To achieve robust transgene induction and minimize secondary effects from prolonged Tcfap2c induction all further analyses were carried out with iTcfap2c animals treated with 0.5 mg dox i.p. at 0 h, 24 h and 48 h followed by analysis on day 3 (72 h).

### Expression of Tcfap2c in the liver results in increased proliferation, apoptosis and microvesicular steatosis

**Figure 3:**
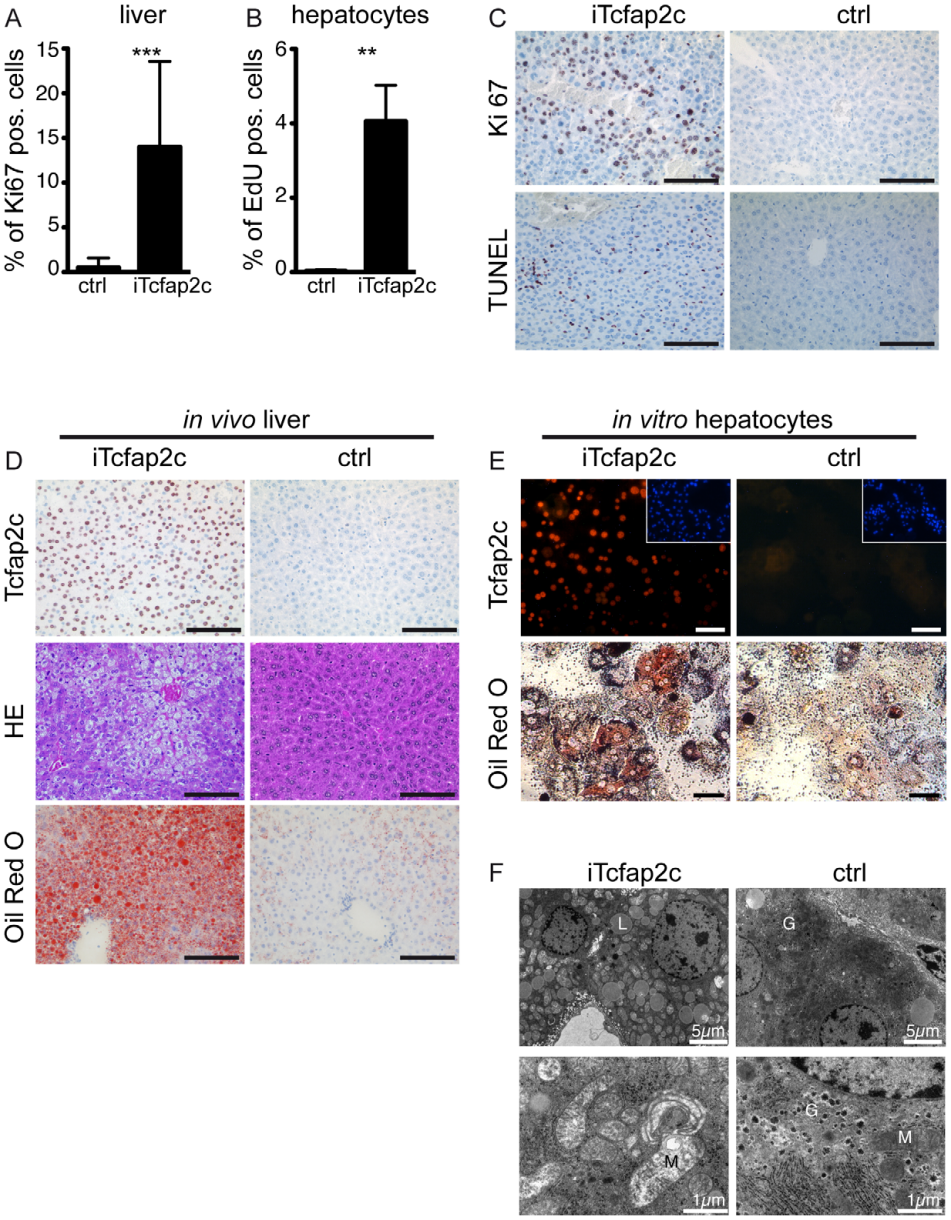
Tcfap2c increases proliferation and induces hepatic steatosis. (A) Percentage of proliferating Ki67 positive hepatocytes in iTcfap2c (n = 5) and ctrl mice (n = 4) (B) Percentage of proliferating hepatocytes in cell culture, as the number of EdU positive cells. Error bars represent standard deviations. ***p≤0.0001, **p≤0.001 (C) Immunohistochemical staining of liver for Ki67 (proliferation, upper panel) and TUNEL staining positive (apoptosis, lower panel). (D) HE (middle panel) and Oil Red-O (lower panel) staining showing fat accumulation and microvisicular steatosis. (E) Tcfap2c immunohistochemistry (upper panel) and Oil-Red O staining (lower panel) of primary hepatocytes. (F) Transmission electron microscope images of iTcfap2c and ctrl liver. Hepatocytes from iTcfap2c mice are filled with lipid droplets (L); Dark and dense mitochondria in ctrl cells but bright mitochondria in iTcfap2c cells were evidence for mitochondria damage. Glycogen granules (G) were visible only in ctrl cells. Scale bars = 100 µm.

The immunohistochemical analyses demonstrated strong induction of Tcfap2c in hepatocytes and the serum analysis indicated liver failure. Since Tcfap2c has been described to affect cellular proliferation as well as cell death [8], [32], we analyzed cellular proliferation and apoptosis. In liver sections of iTcfap2c animals the number of proliferating cells was significantly increased (14.0%) when compared to control sections (0.5%) (Fig.3 A). To analyze whether the activation of proliferation is a cell-intrinsic effect caused by upregulation of Tcfap2c in these cells, we established an in vitro culture of primary hepatocytes from iTcfap2c livers. The cultures were treated with dox for 3 days in order to compare the results with the in vivo situation. In addition, primary hepatocytes isolated from iTcfap2c animals showed significant induction of proliferation (Fig.3 B). These results indicate that increased proliferation is a cell intrinsic primary effect caused by Tcfap2c expression in vitro and in vivo.

Upon close examination an increased number of cells with condensed cytoplasm were detected in the liver of iTcfap2c mice after dox-treatment. Most of those cells were positive in TUNEL assay indicative for apoptosis (Fig.3 C) Hence, ectopic expression of Tcfap2c led to an increase in both, proliferation and apoptosis.

In addition, morphological and metabolic changes were observed. Most hepatocytes of iTcfap2c animals displayed vesicular bodies indicative for microvesicular steatosis (Fig.3 D). This condition (characterized by cytoplasmic fat vesicles (liposomes) that do not displace the nucleus) was detected in more than 50% of the hepatocytes using Oil-Red-O staining (Fig.3 D). The accumulation of fat was also detected in iTcfap2c primary hepatocytes (Fig.3 E). In addition, transmission electron microscopy analysis of the liver showed lack of glycogen storage in iTcfap2c mice compared to controls. Furthermore mitochondria were abnormally bright and increased mitophagy was observed indicating mitochondrial damage. Since impaired mitochondria are no longer able to perform beta-oxidation of fatty acids in the mitochondrial matrix, this helps in explaining the observed steatosis (Fig.3 E).

### Gene array analysis reveals metabolic changes in hepatic lipid and glucose metabolism

**Figure 4:**
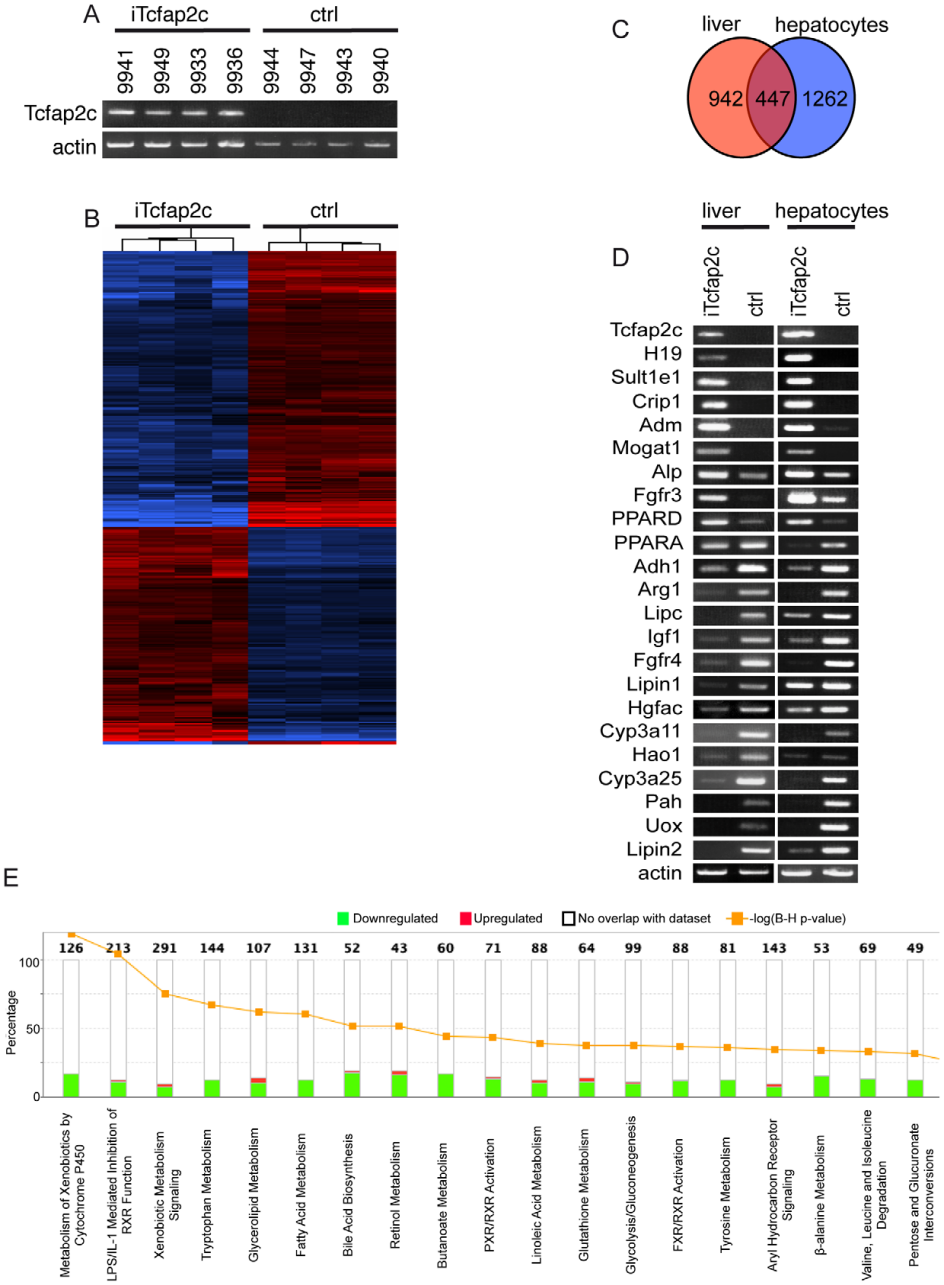
Genes regulated by Tcfap2c and deregulated pathways. (A) Affymetrix microarray gene expression analysis performed with RNA extracted from livers of iTcfap2c (n = 4) and ctrl (n = 4) mice. (B) Heat map showing genes with at least twofold difference between any pair of samples from different classes. Red and blue indicate higher and lower relative expression, respectively. (C) Venn diagram; commonly regulated transcripts by Tcfap2c in vivo and in vitro are shown in the intersection part. (D) RT-PCR of RNA isolated from iTcfap2c and control liver (left column) and Hepatocytes (right colum using the genes indicated (E) Top 18 most significantly regulated pathways are presented based on common gene lists regulated by Tcfap2c.

To determine which genes were differentially expressed upon Tcfap2c-induction microarray analysis was performed using liver samples (n = 8) (Fig.4 A). Unpaired t-test with a p-value cut-off of 0.05 and a 2-fold gene transcript change were used as filters. A total of 1389 differentially expressed transcripts was identified and is shown in a hierarchical cluster heat map (Fig. 4 B, Full gene list Table S6). Next, the gene expression profile from iTcfap2c-liver tissue was compared to the gene expression profile of *in vitro* cultured iTcfap2c- hepatocytes. Analysis of both expression profiles revealed a set of 447 common genes, which are deregulated in both liver and hepatocytes as shown in the Venn diagram (Fig. 4, C). We reasoned that these 447 genes represent the genes directly affected by the upregulation of Tcfap2c. Hence further analyses were carried out using the 447 commonly deregulated genes. We next addressed the question, whether this set of genes could be involved in the pathomorphological changes i.e. the steatosis, proliferation and apoptosis in the liver of iTcfap2c mice. Ingenuity Pathway analysis (IPA) software was used to assist in biological interpretation of the deregulated genes. Interestingly, the gene-set clustered to functional categories regulating ‘lipid metabolism’, ‘hepatic systemic diseases’ and ‘other metabolic processes’ (Table S2). The downregulation of these metabolic pathways is in line with the steatosis observed after induction of Tcfap2c (Fig.4 E, Table S3). The canonical pathway most significantly affected (-log 11.072) was *‘metabolism of xenobiotics by cytochrome P450’* (Fig.4 E, Table S3). The cytochrome P450 (CYP) enzyme system is crucial for the metabolism of xenobiotics as well as endogenous substances, such as fatty acids, prostaglandins and steroids [33]. Of note, more than half of the regulated pathways are linked to lipid metabolism processes and, as indicated by green bars, most involved genes were downregulated. Also, genes associated with glycolysis and gluconeogenesis were affected, explaining the loss of glycogen granules after induction of Tcfap2c (Fig.4 E). Interestingly, RXR mediated pathways were three of the top most significant canonical pathways deregulated by Tcfap2c: ‘*LPS/IL-1-mediated inhibition of RXR-function’*, ‘*PXR/RXR-activation’* and ‘*FXR/RXR-activation’*. This suggests a feedback loop, since Tcfap2c has been reported to be induced by retinoic acid [3], [6].

Next, we generated statistic gene networks, reflecting possible direct and indirect gene interactions for the common gene list. IPA analysis detected 13 highly scoring networks (Table S4). Six are linked to lipid metabolism but there are also connections to cell death, cell cycle, cellular development, cellular growth and proliferation. Subsequently, differential expression of genes encoding metabolic enzymes (Sult1e1, Adm, Mogat1, Adh1, Arg1, Cyp3a11, Cyp3a25, Hao1, Pah, Uox, Lipc, Lipin1, Lipin2) as well as the key regulators of lipid metabolism (peroxisome proliferator-activated receptor alpha and delta, PPARA, PPARD) were verified by RT-PCR in liver tissues as well as in hepatocyte cultures of iTcfap2c mice (Fig.4 D). All selected genes were deregulated in agreement with the array analysis.

Furthermore, Tcfap2c expression affects various signaling cascades via transcriptional regulation of Fgfr3, Fgfr4, insulin-like growth factor (IGF1) and hepatocyte growth factor activator (Hgfac). Additionally, Tcfap2c induced the expression of cancer-associated markers such as, CRIP1 a putative target gene of TFAP2 in prostate cancer [34]. Also, the imprinted, maternally expressed and non-protein coding transcript H19, which is highly expressed in liver metastasis and other tumors was upregulated upon activation of Tcfap2c (see Table S6). Thus, these data provide molecular evidence for the oncogenic potential of Tcfap2c observed in other studies [35].

To further understand the transcriptional networks controlled by Tcfap2c we asked, whether the promoters of the 447 candidate genes described here were bound by Tcfap2c. We performed a meta-analysis on the genome-wide Chromatin-IP chip (ChIP-chip) dataset of Tcfap2c occupied regions in trophoblast stem cells [36] and detected an overlap of 77 genes, from which 47 were found activated and 30 were repressed in our gene arrays (Table S7). Further analysis using TRANSFAC software revealed, that 20 of these genes had bona fide Tcfap2c binding sites within the promotor region and represent the most likely candidates for a direct regulation by Tcfap2c (Table S7, black). These genes were (upregulated) Tcfap2c, Crip1, Hsbp1, Plp2, Crip2, Pdlim1, Jup, Midn, Sox9, Vcl, PPARD, Ralgds, JunB, Rgl3, Tgfrbr1, Gab2 and (downregulated) Spn3, Fads2, Reep6, Fgfr4. These data provide a framework for further understanding of Tcfap2c regulated networks in development and disease.

### Tcfap2c in the intestine led to changes in proliferation and differentiation

**Figure 5:**
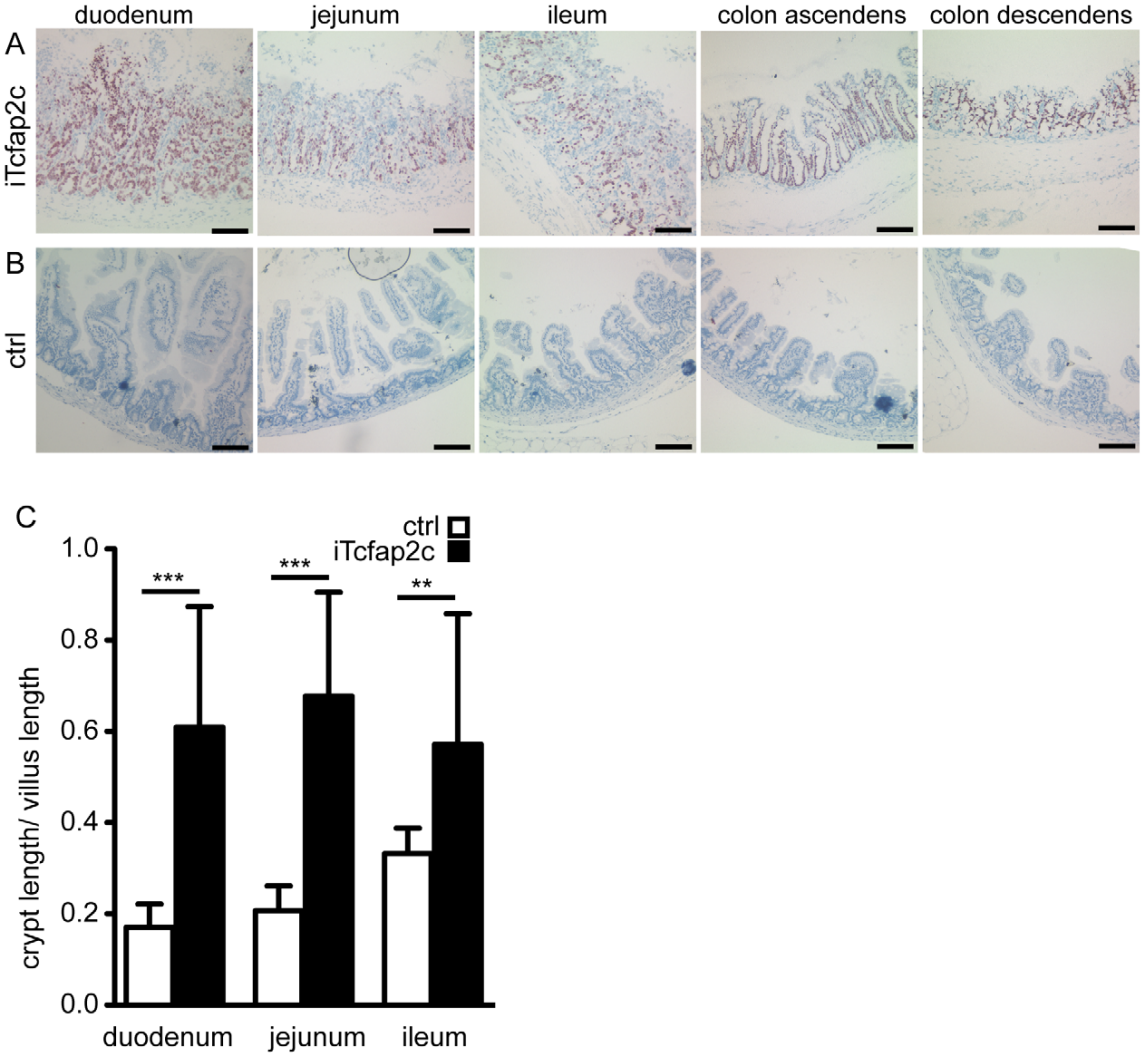
Tcfap2c expression leads to dysplastic growth in the intestine. Immunhistochemical detection of Tcfap2c expression on intestinal sections of iTcfap2c (A) and control mice (B). (C) Ratio of crypt length/villus length the area indicated. Length of villus was measured as the distance from the intestinal muscularis mucosa to the villus-apex; length of the crypts was measured as distance from muscularis mucosae to the bottom of the villus. ***p≤0.0001, **p≤0.001, Scale bar = 100 µm.

**Figure 6:**
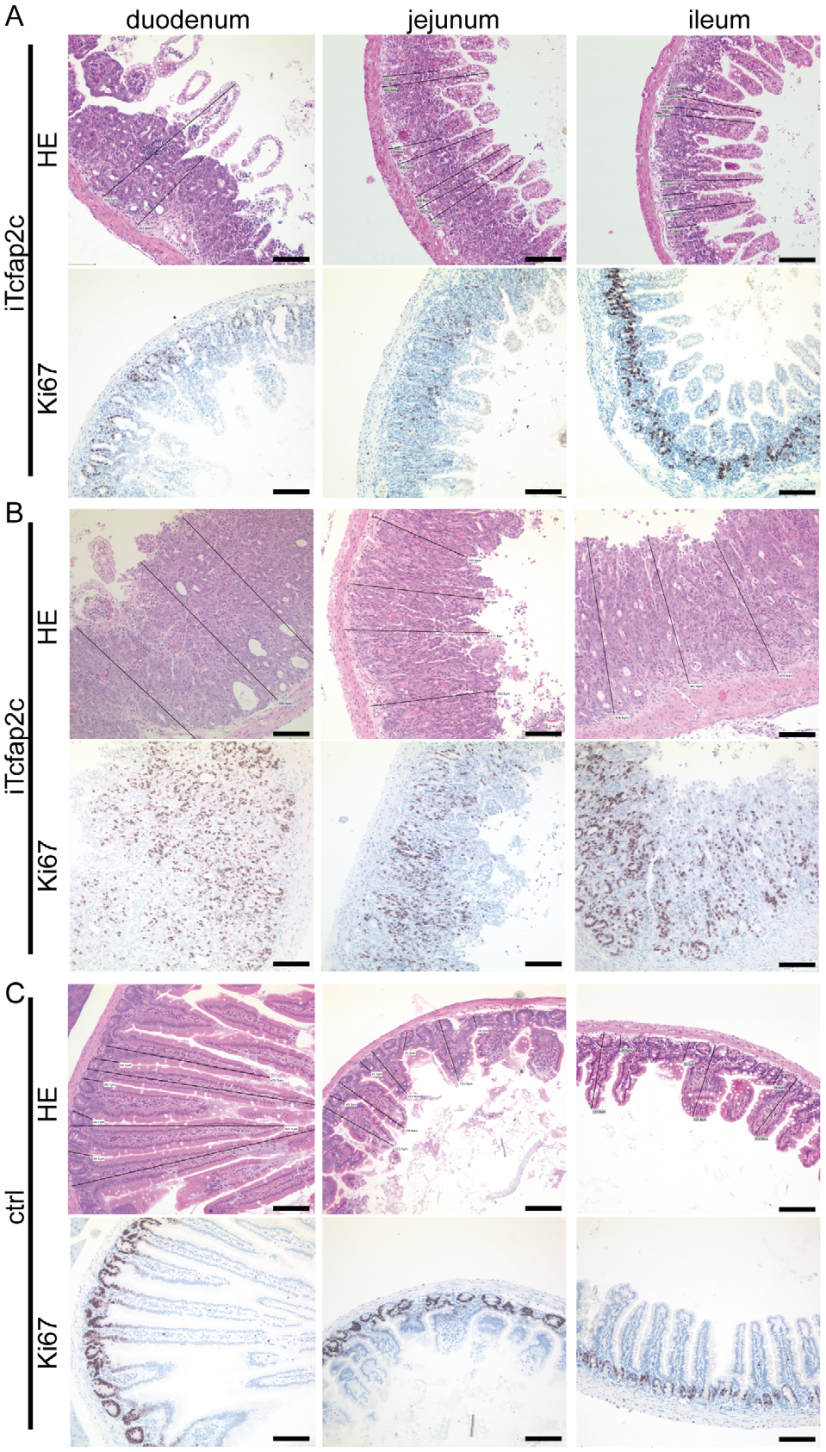
Tcfap2c induces proliferation of the intestine. Sections of the intestine of iTcfap2c (A,B) and control (C) showing HE (upper panel) and Ki67 staining. Scale bars = 100 µm.

Aside from the liver, the transgene was expressed in epithelial cells of the crypts and villi of the intestinal lamina propria mucosae, but not in the mesenchymal cells and the lamina muscularis mucosae (Fig.5 A). Three days after Tcfap2c-induction, the epithelial cells of the intestinal mucosa showed dysplastic growth (Fig.5 A, B). The cells appeared morphologically immature. The crypt-depth to villus-height was measured and this ratio was significantly shifted from approx. 0.2 to 0.6 in iTcfap2c-mice indicating strong hyperplasia (Fig.5 C). Furthermore, Ki67 staining revealed that the proliferative zone, which is usually restricted to the base of the crypt, expanded to cover crypt and villus upon induction of Tcfap2c. The progress and severity of proliferation varied within the animals analyzed (Fig.6 A, B) but could always be considered as hyperplastic when compared to control animals (Fig.6 C).

**Figure 7:**
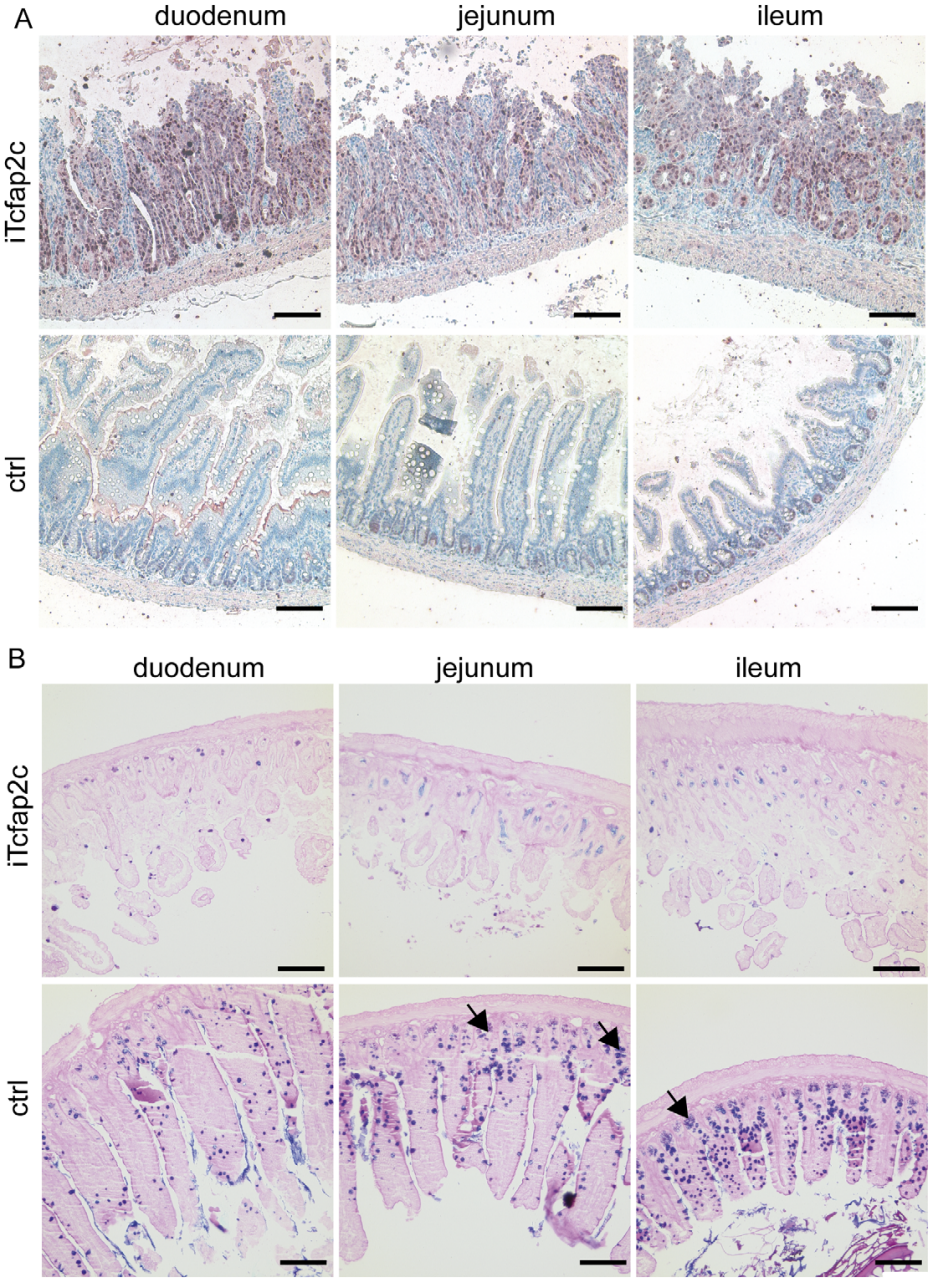
Induction of Tcfap2c results in expansion of the progenitor compartment. (A) Sox9 staining of the intestine iTcfap2c (upper panel) and controls (lower panel). (B) Alcian blue-PAS staining of the intestine iTcfap2c (upper panel) and controls (lower panel). Arrowheads indicate Paneth cells, found at the bottom of the crypts by blue coloring of their granules. Scale bar = 100 µm.

We next addressed the question whether the proliferating cells represent immature (progenitor cells) or differentiated cells, which were forced back into cell-cycle. In the intestine, the progenitor cells, which are responsible for constant renewal of the intestinal tissue display high Ki-67 activity and express the HMG-group transcription factor SOX9 [37], [38], [39]. Immunohistochemical detection of Sox9 showed that all hyperplasic cells were positive for Sox9 (Fig.7 A) suggesting an expansion of the progenitor compartment. In parallel, the number of terminally differentiated derivatives was dramatically reduced (absorptive enterocytes, goblet cells, Paneth cells and enteroendocrine cells) as shown by Alcian blue/periodic acid Schiff (PAS) staining (Fig. 7 B). So, Tcfap2c mediated activation of Sox9 in liver and intestinal cells might inhibit differentiation and induce a cell-type specific progenitor program.

## Discussion

Here, we generated a mouse model in which Tcfap2c could be activated in an inducible and reversible manner by doxycycline in a variety of tissues. Induction of transgenic Tcfap2c in adult mice resulted in rapid lethality within 3–7 days depending on dox concentration. The mice displayed liver steatosis resulting in systemic organ failure and subsequently death of the animal. Expression array experiments revealed that the induction of Tcfap2c in the liver resulted in dramatic downregulation of pathways responsible for lipid metabolism, which provides a link to the molecular mechanism underlying the pathomorphological phenotype. In the intestine, induction of Tcfap2c led to a dramatic expansion of the progenitor compartment (hyperplasia) and concomitant loss of terminally differentiated cells.

The expression and induction pattern of the Tcfap2c transgene closely mirrors the results reported from Oct4 [40] and EGFP-inducible [41] transgenic mice, all taking advantage of the same doxicycline-dependent and Rosa26-promotor driven rtTA-system. Hence, the differences in induction of the transgene in the various tissues are most likely due to the system used and not a result of a specific role of the respective transgene.

The most striking pathomorphological effect of iTcfap2c-induction was observed in the liver. Hepatocytes displayed an altered proliferation, morphology and metabolism. The high serum concentrations of liver specific enzymes and the increased number of apoptotic hepatocytes are indicative of liver damage. Increased proliferation of the liver could be a compensatory effect, due to acute liver damage and cell death of hepatocytes suffering from lipid accumulation and increased oxidative stress. Since this increase was also seen in primary hepatocytes, induction of proliferation is most likely a cell intrinsic consequence of Tcfap2c expression.

Hepatocytes showed a fast occurring and strong microvesicular steatosis by accumulation of lipids, indicating a shutdown of metabolic enzymes. Gene expression further corroborated this finding. Tcfap2c expression significantly influenced the expression of genes that play a role in gylcerolipid metabolism, fatty acid metabolism, xenobyotic metabolism as well as glycolysis as shown by pathway analysis. In addition, the absence of glycogen together with mitochondrial abnormalities detected by electron microscopy, were further hallmarks of impaired lipid beta-oxidation. Further, downregulation of SDHC (succinate dehydrogenase complex, subunit C, integral membrane protein) as well as mitochondria associated cytochrome P450 enzymes (like Cyp27a1) suggests that the observed phenotype might be caused in part by dysfunctional mitochochondria.

The repression of a variety of cytochrome P450 (CYP) enzymes after Tcfap2c induction strongly indicates the inhibition of omega-oxidation processes. In addition, inhibition of CYP enzymes has been reported in livers of patients with steatosis [42]. The substrates of CYP enzymes include metabolic intermediates such as lipids and steroidal hormones, as well as xenobiotic substances. The pathway of the ‘xenobiotic metabolism by cytochrome P450’ is downregulated and is connected to the regulation of peroxisome proliferator-activated receptor alpha (PPARA), and PXR receptors. PPARA and the metabolic co-regulators (Lipin1, Lipin2) were downregulated as well. Reduction of PPARA and Lipin1-2 decreases the hepatic capacity for fatty acid oxidation leading to steatosis [43], [44]. Further, PPARA regulates genes encoding peroxisomal, microsomal and mitochondrial fatty acid metabolizing enzymes in the liver [45].

Due to the rapid death of the iTcfap2c-mice cellular transformation was not observed. However, tumor-associated genes were found up-regulated in iTcfap2c-mice. H19, an imprinted gene that displays maternal monoallelic expression during development and is reactivated during adult tissue regeneration and hepatocellular carcinoma [46], [47] was induced. Also cysteine-rich intestinal protein 1 (CRIP1), an early marker of mammary carcinoma [48] was up-regulated as a consequence of Tcfap2c upregulation. So it seems conceivable, that sub-lethal chronic and/or tissue specific induction of Tcfap2c might eventually result in oncogenic transformation.

Furthermore, ectopic expression of Tcfap2c in adult mice resulted in dysplastic growth of the epithelial cells of the mucosa in the intestinal tract. There, induction of Tcfap2c resulted in an expansion of the proliferative zone in the intestine. In parallel, we observed the lack of terminally differentiated cells in the intestine after induction of Tcfap2c indicated by the disappearance of PAS positive cells. This phenotype can be obtained by two mechanisms i) expression of Tcfap2c re-induces proliferation in differentiated gut mucosal epithelial cells or ii) induction of Tcfap2c leads to expansion of the undifferentiated progenitor cells and repression of terminal differentiation. While scenario i) cannot be ruled out formally, we favor scenario ii) the expansion of the progenitor compartment, since virtual all proliferative intestinal epithelial cells display immunoreactivity for Sox9, a marker for immature progenitor cells [37].

How might Tcfap2c shut off the differentiation pathways in liver and intestine? The meta-analysis, which demonstrated that 20 of the deregulated genes harbor bona fide Tcfap2c binding sites (and are bound by Tcfap2c) indicated that the SRY-box containing gene 9, (Sox9) is a gene transactivated by Tcfap2c. Sox9 lineage tracing showed that adult intestinal cells, hepatocytes and pancreatic acinar cells are arising from Sox9-expressing progenitors [39]. Furthermore, adult hepatic stem/progenitor cells are described to express Sox9 and seem to be the cellular source for liver regeneration [49]. So, Tcfap2c mediated activation of Sox9 might induce the cell-type specific progenitor program in liver and intestinal cells, leading to the inhibition/downregulation of the differentiation markers. In addition, IPA analysis identified novel transcription regulatory networks affecting proliferation and cell death.

The role of Tcfap2c in repressing differentiation and supporting proliferation has been reported in several systems. Tcfap2c helps to maintain the undifferentiated state of primordial germ cells by repressing mesodermal differentiation [15]. Additionally Tcfap2c is highly expressed in undifferentiated germ cell tumors (carcinoma in situ and seminoma) and becomes downregulated in differentiated germ cell tumors such as teratocarcinoma [22], [23]. In early embryonic development Tcfap2c is essential for trophoblast stem cell self-renewal and deletion of Tcfap2c leads to a reduced proliferation and premature differentiation of extraembryonic tissues [9], [14]. Increased levels of Tcfap2c are detected in poorly differentiated breast tumor samples [17]. MMTV-driven transgenic overexpression of Tcfap2c in the mammary epithelium leads to increase in proliferation and an impaired differentiation [32]. Of note, the cell cycle regulator and human TFAP2C target gene CDNK1A/p21 [27], [29] was not isolated in our system. This could be due to epigenetic modifications restricting access to the p21 locus or a lack of appropriate co-factors in the tissues analyzed. Taken together we propose the following model: Induction of Tcfap2c in the liver leads to downregulation of metabolic pathways, i.e. indicators of terminally differentiated cells. This dedifferentiation leads to an induction of the proliferation. We hypothesize, that this is not being tolerated by the system and results in death of the cells and subsequently liver failure. In the intestine on the other hand we observe a dramatic expansion of the progenitor compartment highlighted by Sox9 expression. The turnover of intestinal ephithelial cells is 3 days, so in iTcfap2c mice the terminally differentiated cells of the villus are replaced by immature progenitors from the crypt during this period of time. These results further substantiate the role for Tcfap2c in supporting proliferation and repressing differentiation in cellular compartments, representing immature/progenitor cells.

## Methods

### Animals

All experiments were were conducted according to the german law of animal protection and in accordance with the approval of the local institutional animal care committees (Landesamt für Natur, Umwelt und Verbraucherschutz, North Rhine-Westphalia, approval ID: #8.87-50.10.31.08.238). All efforts were made to minimize the number of animals used and their suffering.

### Generation of transgenic mice

Doxycycline-inducible Tcfap2c ESC cells [tg(Col1a1::TetO-Tcfap2c)(R26::rtTA)] are described previously [14]. To generate chimeric mice, C57BL/6J blastocysts injected with Tcfap2c inducible ES cells were transferred into pseudopregnant foster mice. Offspring of germ line-transmitting chimeric mice were screened for the presence of the targeted ROSA26 and Col1a1 allele by PCR. The mice were registered with the mouse genome informatics database, the allele was named Col1a1 <tm1(tetO-Tcfap2c) Hsc> (MGI:4949113).

### Transgene induction

Experiments were carried out on adult mice (C57BL/6J x 129/Sv) at 8–16 weeks of age. Mice were administered either 0.1 mg/ml doxycycline (Sigma) in the drinking water supplemented with 10% succrose or by intra peritoneal (i.p.) injection of 1 or 0.5 mg doxycycline dissolved in PBS. For cultured cells, doxycycline was used at a concentration of 1 µg/ml.

### Histological analysis

Tissue samples from double transgenic (iTcfap2c) and control animals were either frozen in liquid nitrogen or fixed in 4% buffered formalin overnight and embedded in paraffin. Frozen tissue sections (10 µm) were incubated in 60% isopropyl alcohol, stained for 10 minutes in Oil-Red-O solution and counterstained with hematoxylin. Formalin fixed sections (2–5 µm) were stained with hematoxylin and eosin (H&E) or Alcian blue/periodic acid Schiff (PAS) to detect tissue proteoglycans. To detect apoptotic cells, deoxynucleotidyl transferase dUTP nick end labelling Kit (ApopTag®Peroxidase Kit, Chemicon/Millipore) was used. For immunostaining, sections were incubated (4°C, overnight) using the following primary antibodies: Tcfap2c (6E4; 1∶500; Abcam), Ki-67 (1∶200, DAKO), Sox9 (1∶200 R&D Systems) and subsequently with biotinylated secondary anti-mouse-antibodies (DAKO) for 30 min., followed by incubation with avidin-coupled peroxidase (Vector Laboratories) for 1 h. Sections were photographed using Diskus software.

### Morphometric analysis of the intestine

Intestine was dissected and fixed with 4% buffered formalin for 24 h. Parts of the intestine (duodenum 1 cm, and jejunum 5 cm distal to the pyloric sphincter; ileum 1.5 proximal to the ceacum) were fixed for another 24 h and subsequently paraffin embedded. Transverse tissue sections were stained with H&E. From each section the depth of at least 4 crypts (from the muscularis mucosa to the bottom of the villus) and the overall height of the corresponding villi (from muscularis mucosa to the villus apex) were determined, to analyze the cryt/villus ratio.

### Primary hepatocyte cell culture

ITcfap2c and control mice were anesthetized and livers were perfused via the portal vein first for 10 minutes with Hanks buffered saline (Gibco), without calcium or magnesium, supplemented with 100 mM EGTA and 5000 U Heparin and following for 10 minutes with 0,05 % collagenase (Worthington) in Williams Medium E (Gibco) supplemented with CaCl2 (2.5 M) and Gentamycine (0.1 mg/ml). Livers were removed, mechanically disrupted, filtered through a mesh (300 µm) and centrifuged at 300 rpm for five min. Vital cells were separated using Percoll gradient centrifugation, washed once with Gey's balanced salt solution (GBSS) and platet on collagenated 24 well-Plates (10^5^ cells/well). ITcfap2c and control cells were treated with doxycycline (1 µg/ml) and expression of Tcfap2c, proliferation and fat accumulation were analyzed after different incubation times.

### Microarray procedures

The microarray study was carried out using Affymetrix Mouse Gene ST 1.0 arrays (Affymetrix, Santa Clara, CA). Briefly, total RNA was extracted from cells with RNAeasy kit including DNAse digestion (Qiagen, Hilden, Germany). RNA integrity was assessed using the Agilent 2100 Bioanalyzer (Agilent Technologies, Böblingen, Germany). For the Affymetrix array, 300 ng of total RNA were used. The arrays were washed and stained according to the manufacturer's recommendations and finally scanned in a GeneChip scanner 3000 (Affymetrix). Normalization was calculated with RMA algorithm implemented in GeneSpringGX software (Agilent Technologies, Böblingen, Germany). We used free trial of Ingenuity software to analyze gene pathways and networks affected by Tcfap2c. The raw microarray data have been deposited in the Gene Expression Omnibus repository (accession number GSE28692), which is a MIAME compliant database.

### RT-PCR

Total RNA was isolated from cells and tissues by using RNeasy Mini kit (Qiagen) according to the manufacturer's instructions. First-strand cDNA was synthesized from 500 ng total RNA using SuperScriptIII reverse transcriptase (Invitrogen) according to the manufacturers instructions. The PCR primer sequences are listed in Table S5.

### Protein analysis

For Western blot analysis, 20 µg of protein extract was run on a 10% polyacrylamide separating gel and blotted onto a PVDF (polyvinylidendifluorid) membrane. Antibodies against the following proteins were used Tcfap2c (1∶500; 6E4/4; Abcam), β-Actin (1∶50000; Santa Cruz).

### Cell proliferation analysis

10^5^ primary hepatocytes per 24-well were grown for 48 h in the presence or absence of 1 µg/ml doxycycline in Williams Medium E (Gibco) (supplemented with glutamin, glucose, hepes pH 4, antibiotics, hydrocortisone, insulin, DMSO and inosine). In vitro proliferation was measured after 12 h incubation with EdU and detected with the Click iT®EdU Imaging Kit (Invitrogen) according the manufacturer's instructions and counted in five visual fields. The following antibodies and dilutions were used for further immunofluorescence staining: rabbit antiTcfap2c polyclonal Ab (1∶400; H77; Santa Cruz), Alexa-488 anti rabbit (1∶1000; DAKO) or Hoechst 33342 (1∶2000; Invitrogen) for nuclear staining. Cells were observed using an IM-DRB fluorescent microscope (Leica).

### Blood analysis

Mouse blood composition and blood chemistry were examined 52 h after the beginning of doxycycline treatment (1 mg/day). Blood was collected in EDTA tubes (Sarstedt) and used for complete blood count. Plasma was separated by centrifugation 8000 rpm and 4°C with “serum gel” tubes (Sarstedt) used for biochemical analysis of enzyme activity and detection of non-cellular blood composition.

### Electron microscopy

ITcfap2c and control mice were sacrificed after 48 h doxycycline treatment and liver sections were fixed immediately in an aqueous solution of glutaraldehyde (2.5%) in 0.1 M sodium cacodylate buffer (pH 7.4). Following fixation at 4°C over 2 days, postfixation was started with 2% osmium tetroxide (0.1 M sodium cacodylate buffer) over night at 4°C. Next, tissues were washed two times in isotonic sodium cacodylate buffer (0.1 M; pH 7.4) and dehydrated through a graded series of ethanol, embedding was initiated in Epon 812 with a polypropylene oxide-Epon mixture (1∶1) over night. Polymerization was performed at 70°C for 24 h. Thin sections were cut on a Leica LBK UM IV ultramicrotome to 60 nm and stained with 3.5% uranyl acetate and lead citrate according to standard protocols. Sections were analyzed with a Phillips CM 10 transmission electron microscope and digital picture analysis software (Olympus- SIS).

### Statistical Analysis

Data are expressed as mean±SD. Statistical comparisons were performed using Student's t-test. p<0.05 was considered to be statistically different.

## Supporting Information

Table S1 **Qualitative and quantitative analysis of in vivo expressed Tcfap2c.**
(PDF)

Table S2 **IPA analysis of significant functional categories based on common genes regulated by Tcfap2c in liver and hepatocyte cultures.**
(PDF)

Table S3 **IPA analysis of regulated canonical pathways based on liver and hepatocyte common gene list.**
(PDF)

Table S4 **IPA analysis of transcriptional gene network regulated by Tcfap2c in induced hepatocytes.**
(PDF)

Table S5 **Primers used for RT-PCR.**
(PDF)

Table S6 **Gene array analysis: List of Tcfap2c deregulated genes in the liver, hepatocyte culture and common deregulated genes.**
(XLSX)

Table S7 **Meta-analysis of the genes detected using the genome-wide Chromatin-IP-chip (ChIP-chip) dataset of Tcfap2c occupied regions in trophoblast stem cells and comparing them to the dataset of 447 differentially expressed genes upon Tcfap2c induction in liver and hepatocytes.** A total of 77 differentially expressed genes were also detected using the ChIP-chip. 47 were induced (red) and 30 were repressed (blue) upon Tcfap2c induction in our experiments. 20 genes display bona fide Tcfap2c binding sites within the promotor as revealed by TRANSFAC analysis (black).
(XLSX)
