# Supplementary material for: Transgenic Overexpression of Tcfap2c/AP-2gamma Results in Liver Failure and Intestinal Dysplasia
Source: PLoS One. 2011 Jul 13;6(7):e22034. doi: 10.1371/journal.pone.0022034 (PMC3135619; doi:10.1371/journal.pone.0022034)
Supplement: Table S1 — Qualitative and quantitative analysis of in vivo expressed Tcfap2c. (PDF) [file pone.0022034.s001.pdf]

Table S1

organ	remarks	control	double transgenic
thymus	all cells	-	+
liver	hepatocytes	-	+++
	endothelial cells	-	-
	bile ducts intrahepatic	-	+
	bile ducts extrahepatic	-	+++
kidney	tubular cortex	-	+
	collecting ducts (medula)	-	+
	interstitial cells (medulla)	-	-
heart	myocytes	-	+
	interstitial fibroblasts	-	+
	endothelial cells	-	+
	pericardial cells	-	+
intestine (small)	crypt cells	-	+++
	villi	-	+++
	mesenchymal cells	-	-
intestine (large)		n.d.	+++
stomach		-	+++
pancreas	islet cells	-	+++
	acinar cells	-	+
lung	bronchial epithelial cells	-	++
	alveolar cells	-	+
	interstitial fibroblasts	-	+
	endothelial cells	-	-
skin	epithelial (epidermis)	-	+++
	fibroblast (dermis)	-	++
	adipocytes	-	+
muscle		-	-
bone marrow		-	++
testis	peritubular cells	-	+
	all other	-	-
breast		n.d.	++
uterus	endometrium	n.d.	+++
	myometrium	n.d.	+
ovary		n.d.	+

Quantification of Tcfap2c induction in various organs (left columns) based on immunohistochemical Tcfap2c detection. For this tissue specific analysis double transgenic male and female mice were induced for three days with doxycycline. Expression level was scored using the following schematic:

- no expression

+ <10% of the cells score Tcfap2c positive

++ 10–50% of the cells score Tcfap2c positive

+++ 50–100% of the cells score Tcfap2c positive

n.d. = not determined
