# Supplementary material for: Transgenic Overexpression of Tcfap2c/AP-2gamma Results in Liver Failure and Intestinal Dysplasia
Source: PLoS One. 2011 Jul 13;6(7):e22034. doi: 10.1371/journal.pone.0022034 (PMC3135619; doi:10.1371/journal.pone.0022034)
Supplement: Table S2 — IPA analysis of significant functional categories based on common genes regulated by Tcfap2c in liver and hepatocyte cultures. (PDF) [file pone.0022034.s002.pdf]

Table S2

Significant functional categories based on common genes regulated by Tcfap2c in liver and hepatocyte cultures

Category	B-H p-value	Common regulated genes (red = upregulated, black = downregulated)
Lipid Metabolism	3.9E-12-1.18E-01	PPARA, LIPC, SLC27A2, APOC4, VTN, PLA1A, PLA2G7, ATP8B1, MTTP, POR, FDFT1, SOAT2, PLCE1, ALDH1A1, LPIN1, FGFR4, SULT1E1, BAAT, AMACR, GLYAT, ALDH8A1, SLCO1A2, ACOT12, PLSCR2, ABCB1B, ACADS, PLA2G6, ALDH1A7, GCK, FADS2, SIK1, ACOT4, MLYCD, CYP2B6, CPS1, AGTR1, FADS1, ANGPTL3, ADM, IGFBP4, SLC10A1, APOF, ADH1C, PROX1, ACOT7, MOGAT1, AVPR1A, SGMS1, AADAC, MTOR, IGF1, PPAP2B, AGPAT2, CEBPA, UGT2B17, ADORA1, ACE, UCP2, CYP27A1, COTL1, PPARD, HSD17B7, G6PD, COL4A3BP, UGT2B10, FDPS, GULO, GHR, CYP3A4, ABCB4, SULT1A1, CAT, FKBP4, UGT2B15, G6PC, RDH5, AOX1, DGAT1, LGALS1, ADH4
Small Molecule Biochemistry	3.9E-12-1.18E-01	TGFBR1, LIPC, DPYD, SLC27A2, VTN, APOC4, PLA1A, ATP8B1, POR, LPIN1, HAL, UOX, SULT1E1, BAAT, AMACR, PAH, ACOT12, PLSCR2, ONECUT1, PLA2G6, FADS2, SIK1, CYP2B6, CPS1, EPHX1, ADM, ALDH4A1, SLC10A1, PROX1, AVPR1A, SGMS1, MTOR, IGF1, PPAP2B, CEBPA, ADORA1, UCP2, COL4A3BP, FAH, SLC43A2, GULO, ABCB4, SULT1A1, FKBP4, DGAT1, AOX1, PPARA, HEBP1, PLA2G7, CDA, MTTP, FGFR3, FDFT1, SOAT2, ALDH1A1, PLCE1, FGFR4, GLYAT, ALDH8A1, SLCO1A2, SLC1A1, ABCB1B, ACADS, ALDH1A7, GCK, SLC7A2, ACOT4, PDK2, MLYCD, AGTR1, ANGPTL3, FADS1, ARG1, IGFBP4, APOF, ADH1C, ACOT7, MOGAT1, AADAC, AGPAT2, UGT2B17, SLC25A15, PDK1, ACE, SRC, DPP4, PXN, SLC25A13, HPD, CYP27A1, PPARD, COTL1, AQP11, HSD17B7, G6PD, UGT2B10, SCLY, FDPS, GHR, CYP3A4, CAT, UGT2B15, G6PC, RDH5, GLDC, CYP2A6, A1CF, LGALS1, ADH4
Vitamin and Mineral Metabolism	3.61E-10-1.18E-01	ADM, LIPC, APOF, ADH1C, PROX1, ATP8B1, MTTP, POR, FDFT1, SOAT2, ALDH1A1, UGT2B17, SULT1E1, BAAT, AMACR, CYP27A1, PPARD, G6PD, HSD17B7, ALDH8A1, FDPS, CYP3A4, ALDH1A7, CAT, UGT2B15, G6PC, DGAT1, AOX1, RDH5, CYP2B6, ANGPTL3, ADH4
Hepatic System Disease	1.71E-08-1.18E-01	PPARA, LIPC, DPYD, VTN, SLC17A1, ATP8B1, MTTP, FGFR3, POR, FDFT1, SOAT2, ALDOB, LPIN2, BAAT, PAH, TDO2, SLCO1A2, IFNAR2, VKORC1, EPHA2, AGTR1, EPHX1, SLC10A1, ADH1C, IQGAP1, AVPR1A, CASP6, AADAC, MTOR, JUN, IGF1, TUBA8, STEAP2, ALPL, ACE, SRC, GSTM1, SLC25A13, HPD, CYP27A1, UGT2B10, GHR, ABCB4, SULT1A1, CAT, CRP, UGT2B15, DGAT1, ADH4
Molecular Transport	1.04E-05-1.18E-01	PPARA, LIPC, SLC27A2, APOC4, HEBP1, PLA1A, MTTP, ATP8B1, POR, LIFR, FDFT1, SOAT2, ALDH1A1, LPIN1, FGFR4, SULT1E1, ALDH8A1, SLCO1A2, SLC1A1, PLSCR2, ABCB1B, PLA2G6, GCK, SLC7A2, FADS2, SIK1, CYP2B6, ANGPTL3, KLF15, ADM, SLC10A1, APOF, ADH1C, AVPR1A, SGMS1, AADAC, MTOR, IGF1, SLC2A2, AGPAT2, CEBPA, SLC25A15, ADORA1, ACE, SLC25A13, UCP2, CYP27A1, PPARD, AQP11, G6PD, SLC43A2, GHR, GULO, CYP3A4, ABCB4, CAT, FKBP4, G6PC, RDH5, DGAT1, LGALS1, ADH4
Drug Metabolism	4.73E-05-1.18E-01	ADM, IGFBP4, GSTM5, VTN, ADH1C, POR, GSTM2, ALDH1A1, IGF1, GSTM3 (includes EG:14864), GSTM4, UGT2B17, SULT1E1, GSTA3, GSTM1, ALDH8A1, HSD17B7, ABCB1B, CYP3A4, ABCB4, ALDH1A7, SULT1A1, RDH5, CYP2B6, CYP2A6, ADH4
Endocrine System Development and Function	4E-04-1.18E-01	ADM, IGFBP4, TGFBR1, HSD17B7, NRG4, ONECUT1, POR, IGF1, CYP3A4, ALDH1A7, SULT1A1, UGT2B17, SULT1E1, CYP2B6
Metabolic Disease	7.85E-04-1.18E-01	TGFBR1, LIPC, DDC, TFR2, SLC17A1, MLLT4, ATP8B1, POR, HAL, LPIN1, PAH, AMACR, RNMT, TAT, PLSCR2, ONECUT1, PLA2G6, VKORC1, FADS2, RPS6KA1, CPS1, ALDH4A1, SLC10A1, DAPK2 (includes EG:23604), PROX1, AVPR1A, XYLB, ARHGEF19, MTOR, IYD, IGF1, TUBA8, CEBPA, ADORA1, CLYBL, UCP2, FAH, PGCP, FAM114A1, FGGY, FKBP4, DGAT1, CTH, MYO1E, PPARA, ARHGAP26, HMGCS2, CDA, MTTP, FLVCR1, FDFT1, SOAT2, ALDH1A1, RIPK4, SLC1A1, ACADS, GYS2, TMCC3, GCK, ITIH5, AUH, SAMM50, MLYCD, AGTR1, ARG1, TBC1D22A, PKLR, RAPGEF4, ACOT7, CLIC4, IQGAP1, MCM4, STEAP2, AGPAT2, SLC2A2, SLC25A15, ALPL, ACE, MMP19, TPMT, NDUFAF4, DPP4, SLC25A13, HPD, CYP27A1, PPARD, TCF7L1, SCLY, FDPS, PMP22, GHR, CRP, FAM135A, DBT, G6PC, CYB5A, GLDC, CYP2A6
Amino Acid Metabolism	3.06E-03-1.18E-01	BAAT, SRC, DPP4, PAH, ALDH4A1, PXN, SLC25A13, TGFBR1, HPD, SLC1A1, FAH, SCLY, FGFR3, SLC43A2, MTOR, GHR, HAL, IGF1, SLC7A2, CEBPA, SLC25A15, GLDC, ARG1
