# Supplementary material for: Transgenic Overexpression of Tcfap2c/AP-2gamma Results in Liver Failure and Intestinal Dysplasia
Source: PLoS One. 2011 Jul 13;6(7):e22034. doi: 10.1371/journal.pone.0022034 (PMC3135619; doi:10.1371/journal.pone.0022034)
Supplement: Table S3 — IPA analysis of regulated canonical pathways based on liver and hepatocyte common gene list. (PDF) [file pone.0022034.s003.pdf]

Table S3

Regulated canonical pathways based on liver and hepatocyte common gene list

Canonical Pathways	-log(B-H p-value)	Common regulated genes
Metabolism of Xenobiotics by Cytochrome P450	11,072	GSTA3, GSTM1, GSTM5, CYP2C70, CYP2A12, ADH1C, UGT2B10, CYP2J5, CYP3A43, GSTM2, CYP3A4, GSTM6, GSTM3 (includes EG:14864), UGT2B15, GSTM4, UGT2B17, CYP2B6, ADHFE1, CYP2A6, EPHX1, ADH4
LPS/IL-1 Mediated Inhibition of RXR Function	9,662	PPARA, ALDH4A1, SLC10A1, LIPC, GSTM5, SLC27A2, APOC4, FMO5, HMGCS2, JUN, GSTM2, ALDH1A1, GSTM3 (includes EG:14864), GSTM4, SULT1E1, GSTA3, GSTM1, CYP2A12, ALDH8A1, SLCO1A2, SULT1D1, CYP3A4, SULT1A1, CAT, CYP2B6, CYP2A6
Xenobiotic Metabolism Signaling	6,982	ALDH4A1, CES2 (includes EG:234671), GSTM5, NQO2, FMO5, GSTM2, ALDH1A1, GSTM3 (includes EG:14864), KEAP1, GSTM4, UGT2B17, SULT1E1, GSTA3, GSTM1, CES6, 2210023G05RIK, ALDH8A1, UGT2B10, SULT1D1, ESD, CYP3A4, SULT1A1, CAT, UGT2B15, MAP2K3, CYP2B6
Tryptophan Metabolism	6,26	ALDH4A1, AADAT, TDO2, DDC, CYP2C70, CYP2A12, CYP2J5, ACMSD, CYP3A43, ALDH1A1, CYP3A4, ALDH1A7, CAT, AUH, CYP2B6, AOX1, CYP2A6, KYNU
Glycerolipid Metabolism	5,789	ALDH4A1, DGKD, LIPC, ADH1C, UGT2B10, MOGAT1, LPIN1, ALDH1A1, ALDH1A7, PPAP2B, AGPAT2, DGAT1, LPIN2, ADHFE1, ADH4
Fatty Acid Metabolism	5,604	ALDH4A1, SLC27A2, CYP2C70, CYP2A12, ADH1C, CYP2J5, ACADS, CYP3A43, ALDH1A1, CYP3A4, ALDH1A7, AUH, CYP2B6, ADHFE1, CYP2A6, ADH4
Bile Acid Biosynthesis	4,821	BAAT, ALDH4A1, SOAT2, ALDH1A1, CYP3A4, CYP27A1, ALDH1A7, ADH1C, ADHFE1, ADH4
Retinol Metabolism	4,819	ALDH1A1, ALDH1A7, ALDH8A1, UGT2B15, UGT2B17, UGT2B10, RDH5, ADH4
Butanoate Metabolism	4,095	ALDH4A1, AADAC, ALDH1A1, ALDH1A7, AUH, DBT, SDHC, HMGCL, HMGCS2, ACADS
PXR/RXR Activation	4,068	PPARA, GSTM1, GSTM2, ALDH1A1, CYP3A4, ALDH1A7, G6PC, CYP2B6, HMGCS2, CYP2A6
Linoleic Acid Metabolism	3,642	PLA2G6, CYP3A43, CYP3A4, CYP2C70, CYP2A12, FADS2, CYP2B6, PLA2G7, CYP2A6, CYP2J5, FADS1
Glutathione Metabolism	3,462	GSTA3, GSTM1, GSTM2, GSTM6, GSTM5, GSTM3 (includes EG:14864), G6PD, GSTM4, ANPEP
Glycolysis/Gluconeogenesis	3,451	ALDH4A1, ALDH1A1, PKLR, ALDOB, ALDH1A7, GCK, ADH1C, LRRC16A, G6PC, ADHFE1, ADH4
FXR/RXR Activation	3,433	PPARA, BAAT, LIPC, SLC10A1, ABCB4, PKLR, CYP27A1, FGFR4, G6PC, FOXA3, MTTP
Tyrosine Metabolism	3,364	IYD, HPD, DDC, ADH1C, DBT, TAT, AOX1, ADHFE1, FAH, ADH4
Aryl Hydrocarbon Receptor Signaling	3,223	GSTA3, ALDH4A1, GSTM1, SRC, GSTM5, NQO2, ALDH8A1, GSTM2, ALDH1A1, JUN, GSTM3 (includes EG:14864), GSTM4, HSPB1
β-alanine Metabolism	3,136	ALDH4A1, ALDH1A1, DPYD, ALDH1A7, AUH, MLYCD, UPB1, ACADS
Valine, Leucine and Isoleucine Degradation	3,085	ALDH4A1, ALDH1A1, ALDH1A7, AUH, DBT, AOX1, HMGCL, HMGCS2, ACADS
Pentose and Glucuronate Interconversions	2,951	XYLB, UGDH, UGT2B15, UGT2B17, UGT2B10, ADH4
