# Supplementary material for: Transgenic Overexpression of Tcfap2c/AP-2gamma Results in Liver Failure and Intestinal Dysplasia
Source: PLoS One. 2011 Jul 13;6(7):e22034. doi: 10.1371/journal.pone.0022034 (PMC3135619; doi:10.1371/journal.pone.0022034)
Supplement: Table S4 — IPA analysis of transcriptional gene network regulated by Tcfap2c in induced hepatocytes. (PDF) [file pone.0022034.s004.pdf]

Table S4

Transcriptional gene network regulated by Tcfap2c induction

Top Functions	Molecules in Network (Black = Common regulated genes)	Score	Focus Molecules
Lipid Metabolism, Small Molecule Biochemistry, Carbohydrate Metabolism	ACADS, ACP5, ALDH1A7, ALPL, CREBL2, CRIP2, CYP2B6, FADS1, FADS2, FDPS, FSH, G6PD, GCK, GHR, hCG, Histone h3, IGFBP4, IRAK2, ITGB5, Lh, LRRC32, MAP2K3, MMP19, ORM1, PAH, PKLR, PPAP2B, PPARA, RAB4A, SLC2A2, SQLE, TGFBR1, TGFBR3, TPM1, TPM2	41	30
Cellular Movement, Endocrine System Development and Function, Lipid Metabolism	Akt, Ap1, CEBPA, Creb, DGAT1, DUSP1, ERK, ERK1/2, Estrogen Receptor, FDFT1, FKBP4, Focal adhesion kinase, GSTM4, H19, Hsp90, HSPB1, IGF1, Jnk, JUN, JUNB, LASP1, LDL, LIPC, NUAK1, -, PLA2G7, PLCE1, PXN, RPS6KA1, SFN, SRC, TFAP2C, VCL, VTN	27	23
Lipid Metabolism, Small Molecule Biochemistry, Vitamin and Mineral Metabolism	AADAC, ADH4, ANGPTL3, AP3M1, APCS, CYP2J5, HAL, HAO1, HGFAC, HNF1A, HNF1B, PGCP, PLA1A, SOAT2, SULT1D1, TFR2, TFRC, UGT2B15	20	15
Lipid Metabolism, Small Molecule Biochemistry, Vitamin and Mineral Metabolism	ACMSD, ADH1C, ALDH1A1, BAAT, CES6, CES2 (includes EG:234671), CYP2C9, CYP7A1, DBI, DDC, FOXA1, FOXA2, FOXA3, HMGCS2, HNF1A, HNF1B, HNF4A, LRP5, NR0B2, NR1H4, NR1I2, NR1I3, NR3C1, NR5A2, ONECUT1, PCK1, PKP2, POR, PTMS, STOM, SULT1A1, SULT1E1, TAT, UGT2B17, UOX	18	18
Cell Death, Developmental Disorder, Cell Cycle	ACOT7, ADORA1, AR, CDKN1A, CDKN1C, CREB1, DLG4, DMPK, EGR1, ESR1, FGFR3, FMR1, FOXM1, GPR125, IGF1R, IL6, IQSEC1, KDR, MCM4, MCM7, MDM2, MED1, MIDN, NIPSNAP1, NOTCH1, PIK3R1, PROX1, SFXN1, SIK1, SLC9A3R1, SPTBN2, SRC, TPM1, VEGFA, YY1	15	16
Cellular Development, Cell-To-Cell Signaling and Interaction, Skeletal and Muscular System Development and Function	ADM, BMP2, CASP8, CCL13, CCR5, CRP, CXCL1, CXCL5, CYP2A6, F2, Gsk3, GSTM1, GSTM2, IFNGR1, IL13, INPP5D, KEAP1, Ldh, LIFR, LOC729505, LTB, MTOR, MVP, NDC80, NEDD9, PDLIM2, PLCG2, PSMB9, PSME2, SEPT11, SOX9, SQSTM1, TGFB1, TNF, ZWINT (includes EG:11130)	14	15
Cell Death, Cell-mediated Immune Response, Cellular Development	ABCB4, ADM, AK4, CCL4, CCL13, CD69, CDK2, CDKN1A, CFLAR, CYP27A1, DAPK2 (includes EG:23604), DSG3, DSP, EIF4EBP1, FAM3B, GAB2, HSD17B7, IgG, IL2, JUP, LGALS1, MAPK14, MED1, PAEP, PECAM1, PHLDA2, Pkc(s), PPL, PRDM1, RND3, SPARC, STK17B, TCOF1, WWC2, ZFP36	14	15
Inflammatory Response, Cellular Growth and Proliferation, Antimicrobial Response	ACTN1, AHR, AICDA, ANXA2, ARG1, AUH, CASP1, CASP6, CCL3L3, CD24, CISH, CITED2, CRIP1, CTNNA1, CTSD, EPHX1, GPT2, IFNAR2, IL4, IL5, LPIN2, LTA, LTB, MAF, PDK1, PDLIM1, PMP22, PPARD, PRDM1, SATB1, SLC2A1, SOCS2, TUBA8, UBE2L6, UPB1	13	14
Immunological Disease, Inflammatory Disease, Neurological Disease	AGTR1, ANPEP, CCL5, CCL9, CCL13, CCL3L3, CD97, CDK2, CSF3, CXCL13, DPP4, DUSP1, ELF1, ETS1, ETS2, GTF2B, IFNG, Igm, IL21, IL17A (includes EG:3605), IL2RA, LTA, LTB, MAPK14, MYO1C, PCK1, PIGR, RALGDS, Ras, RNA polymerase II, SLC27A2, SMARCB1, TLR9, TNNI3, TPMT	13	14
Protein Synthesis, Cellular Function and Maintenance, Cell Death	ABCB1B, ACADM, ACE, AGRN, ARPC1B, BAX, CASP1, CAT, CDK2, COL1A1, DDX25, DPYD, DRD2, E2F1, EIF4E, EPHA2, GCG, GRIN2B, GSTM5, HRAS, HSPB1, HTT, IGFBP1, INS, LPIN1, NR1I2, PI3K (complex), SDHC, SLC1A1, SP1, SQSTM1, TAGLN2, TP53, YBX1, ZBTB10	13	14
Lipid Metabolism, Small Molecule Biochemistry, Carbohydrate Metabolism	ACADVL, ALDOB, CDH1, CPS1, CREBBP, CYB5A, CYP2A12, CYP2C70, CYP3A4, EHHADH, EP300, FASN, FMO5, GCN1L1, GSTA3, GSTM6, GSTM3 (includes EG:14864), HADHA, HMGCR, IL6, IMPACT, INS, INS1, IQGAP1, LEP, Mediator, NFE2L2, SC4MOL, SC5DL, SCD, SREBF1, SREBF2, TLE3, VEGFA, ZW10	11	13
Cellular Movement, Hematopoiesis, Immune Cell Trafficking	BIRC2, CCL13, CFLAR, CXCL1, CXCL5, EHD1, ELF3, EPHB2, ERK, HGF, IL6, KYNU, Ldh, LIFR, MAP3K5 (includes EG:4217), MAPK3, Mek, MIF, MMD, NRG1, PI3K (complex), PIGR, PLAU, PTPN12, SAA1, SDC2, SKIL, SLC10A1, SLCO1A2, TAB3, TIAM1, TNF, TRAF2, Vegf, VEGFC	10	12
Lipid Metabolism, Molecular Transport, Small Molecule Biochemistry	ADIPOQ, AKT1, APOB, CCND1, CD36, CDKN1C, CEBPA, CTNNB1, DGAT1, EP300, FASN, FN1, FOXC2, G6PC, HNF4A, ID3, IL6, INS, LDL, LOC100129193, LPL, MEN1, MTTP, NFKB1, NOS2, NR1I2, ONECUT1, PPARG, RPA2, SCARB1, SCD, SGMS1, SLC2A4, STIM1, UCP2	7	10
