# Supplementary material for: Transgenic Overexpression of Tcfap2c/AP-2gamma Results in Liver Failure and Intestinal Dysplasia
Source: PLoS One. 2011 Jul 13;6(7):e22034. doi: 10.1371/journal.pone.0022034 (PMC3135619; doi:10.1371/journal.pone.0022034)
Supplement: Table S5 — Primers used for RT-PCR. (PDF) [file pone.0022034.s005.pdf]

Table S5

Primer sequences

Gene	Primer Sequence (5' → 3')
H19	F: GTCTCGAAGAGCTCGGACTG<br>R: TGACTGCCCTTCTGTCCTCT
Sult1e1	F: CAAAGATGTCGCCGTTTCTT<br>R: TTTTCCCACCAAGCTTTCAC
Crip1	F: TGCGACAAGGAGGTGTATTTC<br>R: GGAGTAGCAGGGATGATTGC
ADM	F: AGCATCCAGCAGCTACCCTA<br>R: ATGCCGTCCTTGTCTTTGTC
Mogat1	F: CTGGTTCTGTTTCCCGTTGT<br>R: TGGGTCAAGGCCATCTTAAC
Alpl	F: GCTGATCATTCCCACGTTTT<br>R: CTGGGCCTGGTAGTTGTTGT
b-Actin	F: CCATCCTGCGTCTGGACCTG<br>R: GTAACAGTCCGCCTAGAAGC
Fgfr3	F: GAGACTTGGCTGCCAGAAAC<br>R: GGAGGACACCAAAAGACCAA
PPARD	F: CTGGAGCTCGATGACAGTGA<br>R: CCGTCTTCTTTAGCCACTGC
PPARA	F: ATGCCAGTACTGCCGTTTTC<br>R: GGCCTTGACCTTGTTCATGT
Adh1	F: ACAAACCCTTCACCATCGAG<br>R: TCGCTTCGGCTACAAAAGTT
Arg1	F: GTGAAGAACCCACGGTCTGT<br>R: CTGGTTGTCAGGGGAGTGTT
Lipc	F: CGGGAAGAACAAGATTGGAA<br>R: TTAAGCCATGCTCTGCAATG
IGF1	F: TGGATGCTCTTCAGTTCGTG<br>R: GTCTTGGGCATGTCAGTGTG
Fgfr4	F: AGCACCCTACTGGACACACC<br>R: TCCGAGGGTACCACACTTTC
Lipin-1	F: CCATTCACAGCGAGTCTTCA<br>R: TGGAAGGGGAATCTGTCTTG

Hgfac	F: CTGGGCGGTTCTGTAACATT<br>R: GGCAATACTCCCACGACAGT
Cyp3a11	F: CAGCTTGGTGCTCCTCTACC<br>R: CTCTGGGTCTGTGACAGCAA
Hao1	F: CTTCGCTGGATGCAACTGTA<br>R: TGCCAAATCATTGGTTTCAA
cyp3a25	F: CCGTTACTTGGCACCATTTT<br>F: GTTTGCCACTGGTGAAGGTT
BMP4	F: GACTTCGAGGCGACACTTCT<br>R: TATACGGTGGAAGCCCTGTT
Pah	F: AGCTTTGCCCAGTTTTCTCA<br>R: TACTCCTGGCAGGCTGTCTT
Uox	F: AGAACACTGTGCACGTCCTG<br>R: CTCATCTGCTCCACCTCACA
Lipin2	F: ACCCAAACCTAGAGCCCTGT<br>R: AATAGAGAGCCGCCACTTCA
